# Mobile phone radiation might alter protein expression in human skin

**DOI:** 10.1186/1471-2164-9-77

**Published:** 2008-02-11

**Authors:** Anu Karinen, Sirpa Heinävaara, Reetta Nylund, Dariusz Leszczynski

**Affiliations:** 1 STUK – Radiation and Nuclear Safety Authority, Laippatie 4, 00880 Helsinki, Finland

## Abstract

**Background:**

Earlier we have shown that the mobile phone radiation (radiofrequency modulated electromagnetic fields; RF-EMF) alters protein expression in human endothelial cell line. This does not mean that similar response will take place in human body exposed to this radiation. Therefore, in this pilot human volunteer study, using proteomics approach, we have examined whether a local exposure of human skin to RF-EMF will cause changes in protein expression in living people.

**Results:**

Small area of forearm's skin in 10 female volunteers was exposed to RF-EMF (specific absorption rate SAR = 1.3 W/kg) and punch biopsies were collected from exposed and non-exposed areas of skin. Proteins extracted from biopsies were separated using 2-DE and protein expression changes were analyzed using PDQuest software. Analysis has identified 8 proteins that were statistically significantly affected (Anova and Wilcoxon tests). Two of the proteins were present in all 10 volunteers. This suggests that protein expression in human skin might be affected by the exposure to RF-EMF. The number of affected proteins was similar to the number of affected proteins observed in our earlier in vitro studies.

**Conclusion:**

This is the first study showing that molecular level changes might take place in human volunteers in response to exposure to RF-EMF. Our study confirms that proteomics screening approach can identify protein targets of RF-EMF in human volunteers.

## Background

Physiological functions of human body are regulated by electric currents. Therefore, is not surprising that placing human body within electromagnetic field, of sufficient strength, may affect physiological processes. The possibility of induction of biological and health effects by low energy radiation emitted by mobile phones (radiofrequency-modulated electromagnetic fields: RF-EMF) remains a controversial issue. In spite of years of research, there is still ongoing discussion whether RF-EMF could induce any physiologically relevant effects [1]. The vast majority of the so far conducted research has focused on cancer. However, RF-EMF is also suspected as potential cause of such ailments as sleep disorders, headaches or allergy-like symptoms [2].

We have proposed that proteomics screening may be used to reveal molecular targets of RF-EMF and help to understand the possible biochemical mechanism of the RF-EMF-induced effects [3]. Our earlier proteomics studies have shown that changes in protein expression and activity (phosphorylation) were induced in human endothelial cell line EA.hy926 that was exposed to RF-EMF [4-7]. These in vitro observed effects, however, do not automatically mean that similar changes would happen in the cells of mobile phone users. Therefore, the present pilot study was undertaken to determine whether a local exposure of human skin to RF-EMF will induce any changes in protein expression and whether it will be possible to find common protein(s) that respond to RF-EMF in all volunteers.

## Results

Ethical permit to perform this study was obtained from the Ethics Committee of Department of Surgery of Hospital District of Helsinki and Uusimaa, Finland. A small skin area of a forearm of 10 of same sex (female) volunteers (age 27 – 65 years; mean 51 years) were irradiated for 1 hour with 900 MHz GSM signal at specific absorption rate (SAR) of 1.3 W/kg, using specially designed exposure setup [8]. The mobile phone safety limit SAR is 2.0 W/kg, as recommended by the International Commission on Non-Ionizing Radiation Protection (ICNIRP).

Immediately after the exposure, a punch biopsy of the exposed area of skin (experimental sample) was taken by a physician. Another punch biopsy was taken from the other, non-exposed, forearm (sham sample). In this experimental set-up each volunteer acted as its own sham control. Both exposed and non-exposed skin samples of all volunteers were immediately snap-frozen in liquid nitrogen and stored before extraction of proteins.

Proteins from all samples were extracted using TRIzol Reagent (Invitrogen) and separated using 2-dimensional gel electrophoresis (2-DE) with pH gradient range of 4–7 in the first dimension and 9% SDS-PAGE gel in the second dimension (GE Healthcare). Proteins were detected by silver staining and spot distribution pattern was analyzed using PDQuest 7.2 software (Bio-Rad).

**Figure 1:**
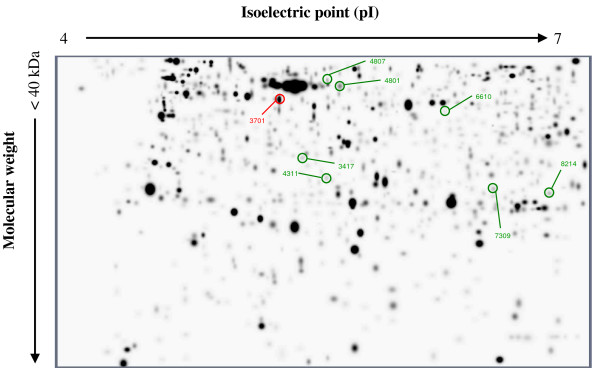
Artificial master gel for all sham and exposed skin samples of all 10 volunteers. Statistically significantly affected protein spots are marked in red color (declined expression) and in green color (increased expression).

We have analyzed a fragment of proteome: proteins with the isoelectric point (pI) 4–7 and the molecular weight <40 kDa, because the protein spot separation in 2-DE in this area was clearly distinguishable (Figure 1). Firstly, using PDQuest software, for each volunteer was generated an artificial gel, by combining protein expression profiles from sham and exposed samples. Thereafter, all 10 artificial gels were combined into single artificial master gel and the differentially expressed protein spots, which were detected in at least 4 volunteers, were statistically analyzed.

The ratio of exposed and sham sample expression was analyzed spot by spot, after logarithm transformation, with variance analysis (anova). Due to small numbers and potential violations of model assumptions, the ratios were also studied with the Wilcoxon test. The statistical analysis has identified 8 differentially expressed proteins where the change in expression was statistically significant among the 579 identified proteins spots (Table 1). Two of the protein spots (#3701, #4801) were present in all 10 volunteers thus showing that it is possible to find common, responding proteins among the all volunteers. The p-values are not adjusted for multiple comparisons.

**Table 1:** List of proteins that were present in at least 4 volunteers and which expression has been changed in statistically significant manner (<0.05) as determined by the variance analysis and the Wilcoxon test. Ratio = exposed sample expression/sham sample expression.

	**Protein spot #**	Prevalence of spot*	**Average of ratio**	**Standard deviation of ratio**	**Median of ratio**	**Anova p-value**	**Wilcoxon p-value**
1.	3417	4	117.35	157.93	1.00	0.0370^+^	0.0474
2.	3701	10	0.68	0.39	0.49	0.0139	0.0367
3.	4311	7	68.88	134.67	1.71	0.0369	0.0169
4.	4801	10	1.89	1.01	1.42	0.0162	0.0218
5.	4807	5	223.17	302.78	1.53	0.0307^+^	0.0277
6.	6610	4	39.86	52.05	1.00	0.0371^+^	0.0474
7.	7309	8	67.38	101.44	12.17	0.0034	0.0076
8.	8214	4	146.60	252.75	1.00	0.0391^+^	0.0474

* prevalence shows in how many volunteers, out of 10, was detected given protein spot;

^+ ^variance analysis might be unreliable because of small numbers of cases and potential violations of model assumptions;

## Discussion

Proteomics approach to study effects of mobile phone radiation on cells has been used so far only by two research groups, ours in Helsinki and group the Zhejiang University, Hangzhou, China. Our studies, using human endothelial cell lines have shown that mobile phone radiation induces statistically significant changes in the expression of several tens of proteins [6] and that the response of cell might be proteome-dependent [7]. In one study, the group in China has not found statistically significant differences in protein expression in MCF-7 cells [9]. The reason for it might be too low number of experiments in MCF-7 study [9]. In our studies statistical analysis was based on 10 different experiments whereas Zeng et al. [9] based their analysis on only three replicates. Another reason for the difference might be different sensitivity of MCF-7 cells as compared with ours endothelial cell lines EA.hy926 and EA.hy926v1. In the other study from Zhejiang University [10] were found 4 differentially expressed proteins in lens epithelial cells, among them the stress response protein Hsp70.

The obtained results, suggesting effect of mobile phone radiation on protein expression in human cell lines, do not automatically mean that this exposure will have any effect on protein expression in humans. The so far conducted human volunteer studies have focused on cognitive responses to RF-EMF [2] and there is no information available about the proteome, as well as transcriptome, response to mobile phone radiation in humans. This study is, to our knowledge, the first one where human response to RF-EMF was examined on molecular level. Our results suggest that human skin might respond to RF-EMF and change protein expression profile. Interestingly, when adjusting results of our previous cellular study [6] using the size of proteome analyzed in the present study (pI 4–7; <40 kDa) the number of the statistically significantly affected proteins appears to be similar in this and in earlier [6] study, 8 spots and 9 spots, respectively. The number of differentially expressed protein spots in both studies is below the number of expected false positives. However, as we have demonstrated experimentally [6] and discussed previously [11] it is likely that some of the proteins will be indeed, real positives. However, without further testing, it is not possible to predict whether these changes will have impact on skin physiology.

Finally, our study confirms that the proposed by us proteomics approach [3] can identify protein targets of RF-EMF. This approach to EMF research has been subsequently accepted by the EMF scientists [12,13] and has been included into the 2006 World Health Organization Research Agenda [14]. However, new and larger study is urgently needed to strengthen our pilot observations and to determine what impact mobile phone exposure might have on human tissues.

## Conclusion

▪ Mobile phone radiation might alter protein expression in human skin.

▪ Physiological significance of this change is not known and requires further study.

▪ Larger human volunteer study will be needed to confirm results of this pilot study.

▪ Proteomics screening is valid method for search for molecular targets of mobile phone radiation. Without this approach the identification of the proteins responding to mobile phone radiation would not be reasonably possible.

## Methods

### Ethical issues

Ethical permit to perform this study was obtained, in accordance with the Helsinki Declaration, from the Ethics Committee of the Department of Surgery of the Hospital District of Helsinki and Uusimaa, Finland (decision #127/2005 issued on November 23, 2005). Each volunteer was informed in detail about all experimental procedures and each of them has signed the informed consent form (in Finnish language).

### Exposure of volunteers to mobile phone radiation

Volunteers were exposed to 900 MHz GSM mobile phone radiation in an experimental setup described in detail elsewhere [8]. The source of irradiation was a half-wave dipole fed with a computer controlled GSM phone. The specific absorption rate (SAR) induced in the skin was 1.3 W/kg what is below the ICNIRP safety guidelines (2.0 W/kg). During the exposure small area of the right forearms was irradiated for one hour. The other, non-irradiated forearm was used as sham control. Immediately after exposure skin punch-biopsies were taken from the exposed and non-exposed skin for protein analysis.

### Protein extraction from skin biopsies

Skin punch biopsies, consisting of both dermis and epidermis but without the underlying fat tissue, were frozen immediately after harvesting in liquid nitrogen and stored at -80°C. Isolation and separation of proteins were performed in the blinded manner. Proteins were isolated from frozen skin using TRIzol^® ^reagent protocol as described by the manufacturer (Invitrogen, Carlsbad, CA, USA) with a few modifications. Briefly, the chopped skin punch-biopsies were immersed in 0.5 ml of ice-cold TRIzol reagent and homogenized on ice with 70 strokes of the pestle in DUALL 1 ml tissue grinder (Kimble Chase Life Science and Research Products, Vineland, NJ, USA). After the phase separation of TRIzol reagent, the organic phase containing DNA and proteins was collected. DNA was then precipitated with ethanol and proteins were isolated from the phenol-ethanol supernatant. The proteins were then precipitated by isopropyl alcohol and pelleted at 12000 × g for 10 min at +4°C. The protein pellet was washed 3 times with 0.3 M guanidine hydrochloride solution in 95% ethanol and once with 99.5% ethanol. During the extraction pellets were grinded with pellet pestle in order to improve the solubility of the proteins. After each wash step, proteins were centrifuged 7500 × g for 5 min at +4°C. The air-dried protein pellet was dissolved in 2-DE rehydration buffer containing 9 M urea, 2% (w/v) CHAPS, 0.5% (v/v) IPG buffer pH 4–7 and 5 mg/ml DTT (added as fresh). The protein concentration of sample was measured using the Bradford method. The samples were stored at -80°C.

### Protein separation with 2-DE

Proteins were separated by standard 2-DE. Briefly, the first dimension was performed in IPGphor™ (GE Healthcare, UK) isoelectric focusing (IEF) apparatus. Linear, 24 cm long, pH 4–7 Immobiline™ DryStrip gels (IPG-strips, GE Healthcare, UK) were rehydrated in the strip holders for 4 hours in 0.45 ml rehydration buffer containing 9 M urea, 2% (w/v) CHAPS, 0.5% (v/v) IPG-buffer pH 4–7, 1.2% (v/v) DeStreak™ reagent, a trace of bromophenol blue and 150 μg of total amount of protein. IEF was carried out at +20°C using following step-and-hold settings: 50 V, 8 h; 100 V, 1 h; 500 V, 1 h; 1000 V, 1 h; 2000 V, 1 h; 8000 V, until 95000 Vh was achieved. Then, the IPG-strips were incubated at room temperature in equilibration buffer (50 mM Tris-HCl, pH 8.8, 6 M urea, 30% (v/v) glycerol, 2% (w/v) SDS, a trace of bromophenol blue, and 10 mg/ml DTT) for 15 min and for another 15 min in the same buffer that contained 25 mg/ml of iodoacetamide instead of DTT. The second-dimension separation was performed using 9%SDS-PAGE gels. Electrophoresis was carried out at +10°C using an Ettan™ DALTsix electrophoresis unit (GE Healtcare) at a constant power of 3.5 W/gel for 0.5 h and then 13 W/gel until the dye front reached the bottom of the gel (about 4 h). The ready gels were silver stained to visualize protein spots. Stained gels were scanned into computer using GS-710 Calibrated Imaging Densitometer (Bio-Rad Laboratories, Hercules, CA, USA). The gels were analyzed using PDQuest 7.2 software (Bio-Rad).

## Abbreviations

CHAPS, 3-[(3-Cholamidopropyl)dimethylammonio]-1-propanesulfonate; 2-DE, 2-dimensional electrophoresis; DTT, dithiothreitol; EA.hy926, human endothelial cell line; ICNIRP, International Commission on Non-Ionizing Radiation Protection; IEF, isoelectric focusing; IPG, immobilized pH gradient; MCF-7, human breast adenocarcinoma cell line; pI, isoelectric point; RF-EMF, radiofrequency modulated electromagnetic field; SAR, specific absorption rate; SDS, sodium dodecyl sulfate; SDS-PAGE, sodium dodecyl sulfate polyacrylamide gel electrophoresis; Tris-HCl, Tris(hydroxymethyl)aminomethane hydrochloride;

## Authors' contributions

AK executed the proteomics experiments and performed analysis of the proteomics data. RN assisted in designing of the study, participated in writing the grant funding the study, assisted in analysis of proteomics data. SH performed statistical analysis of the data. DL conceived and designed the study, obtained grant funding the study, coordinated execution and analysis of the results and wrote the draft manuscript. All authors participated in the writing of the final version of the manuscript, read it and approved it.
